# PGRS Domain of Rv0297 of *Mycobacterium tuberculosis* Is Involved in Modulation of Macrophage Functions to Favor Bacterial Persistence

**DOI:** 10.3389/fcimb.2020.00451

**Published:** 2020-09-11

**Authors:** Tarina Sharma, Sonam Grover, Naresh Arora, Manjunath P, Nasreen Zafar Ehtesham, Seyed Ehtesham Hasnain

**Affiliations:** ^1^Kusuma School of Biological Sciences, Indian Institute of Technology, New Delhi, India; ^2^Institute of Molecular Medicine, Jamia Hamdard, New Delhi, India; ^3^ICMR-National Institute of Pathology, New Delhi, India; ^4^Dr. Reddy's Institute of Life Sciences, Hyderabad, India

**Keywords:** apoptosis, endosomal markers, lung granulomas, *Mycobacterium smegmatis*, PE_PGRS5, phagosome maturation

## Abstract

*Mycobacterium tuberculosis (M. tb)* Rv0297-encoded PE_PGRS5 has been known to be expressed at the later stages of infection and in acidified phagosomes during transcriptome and proteomic studies. The possible role of Rv0297 in the modulation of phagosomal maturation and in providing protection against a microbicidal environment has been hypothesized. We show that Rv0297PGRS is involved in modulating the calcium homeostasis of macrophages followed by impedance of the phagolysosomal acidification process. This is evident from the downregulation of the late endosomal markers (Rab7 and cathepsin D) in the macrophages infected with recombinant *Mycobacterium smegmatis* (r*M.smeg*)—*M.smeg_Rv0297* and *M.smeg_Rv0297PGRS—*or treated with recombinant Rv0297PGRS protein. Macrophages infected with r*M.smeg* expressing Rv0297 produce nitric oxide and undergo apoptosis, which may aid in the dissemination of pathogen in the later stages of infection. Rv0297 was also found to be involved in rescuing the bacterium from oxidative and hypoxic stress employed by macrophages and augmented the survivability of the recombinant bacterium. These results attribute to the functional significance of this protein in *M.tb* virulence mechanism. The fact that this protein gets expressed at the later stages of lung granulomas during *M.tb* infection suggests that the bacterium possibly employs Rv0297 as its dissemination and survival strategy.

## Introduction

Tuberculosis (TB), the leading cause of death worldwide, is caused by *Mycobacterium tuberculosis* (*M.tb*). The World Health Organization (WHO) 2019 report stated ~1.3 million deaths in HIV-negative patients with an additional loss of 300,000 among HIV-positive patients. Around 10 million new cases of TB have been estimated globally. The emergence of multidrug-resistant (MDR) and extensively drug-resistant (XDR) strains has worsened the conditions in the past two decades. India accounts for 24% of the total MDR/XDR-TB cases, followed by 13% in China and 10% in the Russian Federation (WHO., 2019). *M.tb*, an intracellular pathogen, has an essential virulence characteristic of survivability in host macrophages. The detailed mechanism employed by *M.tb* to survive in the highly microbicidal environment of macrophages is very complex and is still enigmatic.

The development and pathogenesis of the disease may depend on several complex networks of host–pathogen interactions within human macrophages. Ca^2+^, an important secondary messenger, is the key molecule that affects the majority of host cellular responses. Several cellular proteins bind to both intracellular as well as extracellular Ca^2+^ ions to modulate downstream signaling within the cell (Clapham, 2007). *M.tb*, an intracellular pathogen, is able to modulate the Ca^2+^ levels within infected macrophages as a vital persistence approach (Vergne et al., 2004). This ability of *M.tb* suggests a crucial link between Ca^2+^ and host cellular cascades—such as interaction with Toll-like receptors (TLRs), immune responses, production of reactive oxygen species (ROS) and nitric oxide (NO) intermediates, apoptosis of host cells, and acidification of phagolysosomes. Calcium signaling is found to be critical for the maturation of phagosomes (Malik et al., 2000, 2001, 2003). Pathogenic mycobacterium possesses the ability to amend the physiological levels of Ca^2+^, thereby hindering the acidification of the phagosomal compartments. This inhibition can also be imitated by the inhibition of calmodulin, sphingosine kinase, or Ca^2+/^calmodulin-dependent protein kinase II (CaMKII) pharmacologically (Malik et al., 2000, 2001, 2003). The acidification process can be re-established by using Ca^2+^ ionophores, which increases the levels of intracellular Ca^2+^ Ions (Malik et al., 2000). *M.tb* blocks the phagolysosomal maturation by not allowing the recruitment of late endosomal markers. Early endosomal markers include transferrin receptor, early endosome antigen 1 (EEA-1), and Rab5 that have to be exchanged by late endosomal markers such as cathepsin D and Rab7. *M.tb* impedes the expression of late endosomal markers, thus decreasing the maturation process of phagolysosomes (Rink et al., 2005; Poteryaev et al., 2010; Thi et al., 2013). Another important aspect in *M.tb* pathogenesis is the involvement of a regulated host cell death in the form of apoptosis, which plays a crucial role in pathogenesis. Though considered as a defense mechanism for the host, apoptosis is now exploited by many pathogenic bacteria, particularly *M.tb*, for their dissemination (Ruckdeschel et al., 1997; Wickstrum et al., 2009). Dissemination of *M.tb* infection *via* the apoptotic bodies has been recently observed (Aguiló et al., 2013).

The PE/PPE/PE_PGRS protein family is coded by 10% of the *M.tb* genome. The PE_PGRS proteins of this family are majorly intrinsically disordered in nature (Cole et al., 1998; Ahmad et al., 2018, 2020; Grover et al., 2018). PE_PGRS proteins are identified as tandem repeats of Gly-Gly-Ala or their variants. Many variations in the size and number of the repetitive sequences of Gly-Gly-Ala or Gly-Gly-Asn motifs are present within different PE_PGRS proteins (Brennan and Delogu, 2002) and are known to serve as a source of antigenic variation (Akhter et al., 2012) and host immune evasion (Brennan and Delogu, 2002; Tiwari et al., 2012, 2014). Multiple functions of the PE_PGRS proteins have been assigned so far, such as host cell apoptosis (Basu et al., 2007; Grover et al., 2018), bacillary survival (Iantomasi et al., 2012), pro- and anti-inflammatory immune responses (Chakhaiyar et al., 2004; Chaitra et al., 2007; Singh et al., 2008; Bansal et al., 2010; Cohen et al., 2014; Khubaib et al., 2016), granuloma maintenance (Ramakrishnan et al., 2000), inhibition of phagosomal maturation (Thi et al., 2013), and resistance to microbicidal components (Singh et al., 2016). Genomic and proteomic differences in several PE_PGRS proteins of *M.tb* H_37_Rv and H_37_Ra have been shown to account for the difference in the pathogenesis and virulence of these strains (Kohli et al., 2012). Evidences supported their role in the virulence and survival of mycobacterium within the host macrophages and granulomas (Ramakrishnan et al., 2000). Dominant and consistent expression of PE_PGRS proteins during TB infection in guinea pig lungs has been shown; specifically, the co-operonic PE-PGRS53/54 and PE-PGRS56/57 proteins have been shown to be among the most dominantly expressed proteins 30 and 90 days post-infection in lung tissues (Kruh et al., 2010). The PGRS domain of PE_PGRS consists of multiple nona-peptide motifs which form a parallel beta helix structure capable of binding with calcium ions (Bachhawat and Singh, 2007). The binding of such proteins may amend the calcium homeostasis or cause a sudden dip in the calcium levels at the focal point of infection and thereby hinder the phagolysosomal acidification and, thus, possibly improve the survival of the pathogen inside macrophages. The involvement of several mycobacterial PE/PPE/PE_PGRS proteins has been identified in arresting the acidification of phagosomes (Stewart et al., 2005). Thus, the role of these PE/PPE proteins in arresting vacuole acidification and consequent maximization of intracellular survival was noticed.

*M.tb* Rv0297-encoded PE_PGRS5 protein has been found to be expressed in lung granulomas 90 days post-infection in a proteomic analysis (Kruh et al., 2010). It was also a part of a *M.tb*-specific genomic island (Becq et al., 2007). In a high-throughput study, enrichment of the *Mycobacterium bovis* Bacille Calmette–Guérin (BCG) PE_PGRS5 mutant in acidified phagosomes was shown (Stewart et al., 2005). The present study has been designed to investigate the likely role of the PGRS domain of *M.tb* Rv0297 in the modulation of calcium homeostasis with subsequent involvement in the impedance of phagolysosomal maturation, modulation of host immune responses, and bacterial persistence *via* the apoptosis of infected host cells. This protein may serve as an important factor in the pathogenesis of tuberculosis and it enhances the surviving capability of mycobacterium. These findings provide better understanding of the pathogenic potential of the PGRS domain of PE_PGRS proteins that can be targeted for therapeutic interventions.

## Materials and Methods

### Generation of Constructs

For generating the rRv0297PGRS protein, the gene coding for Rv0297PGRS was cloned in a pET28a expression vector and a recombinant protein purified as described in our previous study (Grover et al., 2018). The Rv0297PGRS gene cloned in the pET28a expression vector was expressed in BL21(DE3)pLysS cells. The recombinant protein was purified from inclusion bodies by solubilization in 8 M urea in phosphate-buffered saline (PBS, pH 7.5) and on-column renaturation using a urea gradient followed by Ni^2+^-nitrilotriacetic acid (NTA) chromatography. The protein was treated with polymyxin B (Sigma) at 4°C for 2 h.

For the generation of recombinant clones expressed in *Mycobacterium smegmatis*, the gene coding for Rv0297 full length and Rv0297PGRS proteins were cloned in a constitutive expression vector pVV16 and transformed in competent *M. smegmatis mc*^2^*155* by electroporation. Positive transformants were grown in 7H9 medium supplemented with 10% (*v*/*v*) albumin–dextrose–catalase (ADC), 50 μg/ml hygromycin, and 25 μg/ml kanamycin. Restriction digestion and Western blotting confirmed the positive clones.

### Cell Culture

The macrophage cell lines human THP-1 and murine RAW264.7 were maintained in Roswell Park Memorial Institute (RPMI 1640) and Dulbecco's modified Eagle's medium (DMEM) respectively supplemented with 10% fetal bovine serum (FBS, Invitrogen), penicillin (100 IU/ml), and streptomycin (100 μg/ml). The required number of cells was seeded in 6- and 24-well plates depending on the experiment. Cells were either treated with different concentrations of the rRv0297PGRS protein or infected with recombinant *M. smegmatis* (r*M.smeg_pVV16*, r*M.smeg_Rv0297*, and *rM.smeg_Rv0297PGRS*).

### *In vitro* Infection of THP-1 With Recombinant *M. smegmatis*

THP-1 cells (2 × 10^6^ cells/well) were seeded in six-well tissue culture plates. The next day, cells were infected with *M.smeg_Rv0297, M.smeg_Rv0297PGRS*, and *M.smeg_pVV16* (vector control) grown to an optical density (OD) of 0.8 at a multiplicity of infection (MOI) of 1:10 in a BSL 2 facility. After 3 h of infection, the cells were washed with PBS and 5–20 μg/ml gentamycin to kill extracellular bacteria, followed by incubation with complete medium for 24 and 48 h. For Western blot analysis, the infected THP-1 macrophages were incubated for 48 h. For the colony forming unit (CFU) assay, the infected cells were incubated for 24 and 48 h.

### Nitrite Quantitation in Macrophages

RAW264.7 cells were infected with recombinant strains expressing Rv0297 and Rv0297PGRS. After 30 h of infection, the cell-free supernatant (150 μl) was mixed with 50 μl of Griess reagent for 30 min. Nitrite concentration was measured using sodium nitrite as a standard. Plates were read at 540 nm.

### Cytokine Assessment in Macrophages

Cells were either infected with the recombinant strains or treated with the Rv0297PGRS protein (0–10 μg/ml) for 30 h. Bovine serum albumin (BSA, 10 μg/ml) and lipopolysaccharide (LPS, 200 ng/ml) have been used as the negative and positive controls for the assessment of cytokines. The cell-free supernatant was collected and the tumor necrosis factor alpha (TNF-α) and interleukin 12 (IL-12) concentrations were measured using an ELISA Kit (eBIosciences) as per the manufacturer's instructions. Plates were read at 450 nm.

### Calcium Release Assay

THP-1 macrophages were treated with the Rv0297PGRS protein (for 30 h) and stained using Fluo-4 NW dye solution (Molecular Probes, Invitrogen). Calcium influx was measured by reading at 494 nm excitation and 516 nm emission wavelengths.

### Western Blot Analysis

Western blot analyses were performed with anti-Rab5, anti-Rab7 (CST), anti-cathepsin D (Cloud-Clone Corp.), anti-cleaved caspase-3 (Santa-Cruz), anti-PARP-1 (Santa-Cruz), and anti-β-actin (Sigma). Membranes were developed using a chemiluminescent reagent (Thermo Fisher).

### *In vitro* Stress Response Assay

*M.smeg_Rv0297, M.smeg_Rv0297PGRS*, and *M.smeg_pVV16* were grown to an OD of 1.0 and diluted in fresh 7H9 medium supplemented with 10% ADC to obtain an OD of 0.2. The bacterial cells were then seeded in 96-well plates and allowed to grow for the next 24 h. After 24 h of growth, oxidative and hypoxic stress was given by using 1–10 mM of H_2_O_2_ and 1–5 mM of CoCl_2_, respectively. After 24 h, cell viability was assessed using 0.3% Resazurin sodium salt by measuring the spectrophotometric reading at 570 and 600 nm and the survival percentage was calculated.

### Bacterial Survivability Assessment in Infected Macrophages

Phorbol 12-myristate 13-acetate (PMA)-differentiated THP-1 macrophages were infected with recombinant *M. smegmatis* expressing Rv0297 and Rv0297PGRS. After 0, 24, and 48 h, the macrophages were lysed and serially diluted, followed by plating on 7H10 agar plates for growth of the bacterial colonies. The CFU of the bacterial colonies were calculated after 48 h of incubation to assess viable bacteria.

### Statistical Analysis

All data were expressed in the form of mean ± standard deviation (SD) derived from three different groups of independent experiments using GraphPad Prism 6.0 software. A one-way analysis of variance (ANOVA) was performed, followed by Dunett's *post hoc* test in order to calculate the statistical significance at *p* < 0.05.

## Results

### Rv0297PGRS Domain Interferes in the Maturation of Phagolysosomes

**Figure 1:**
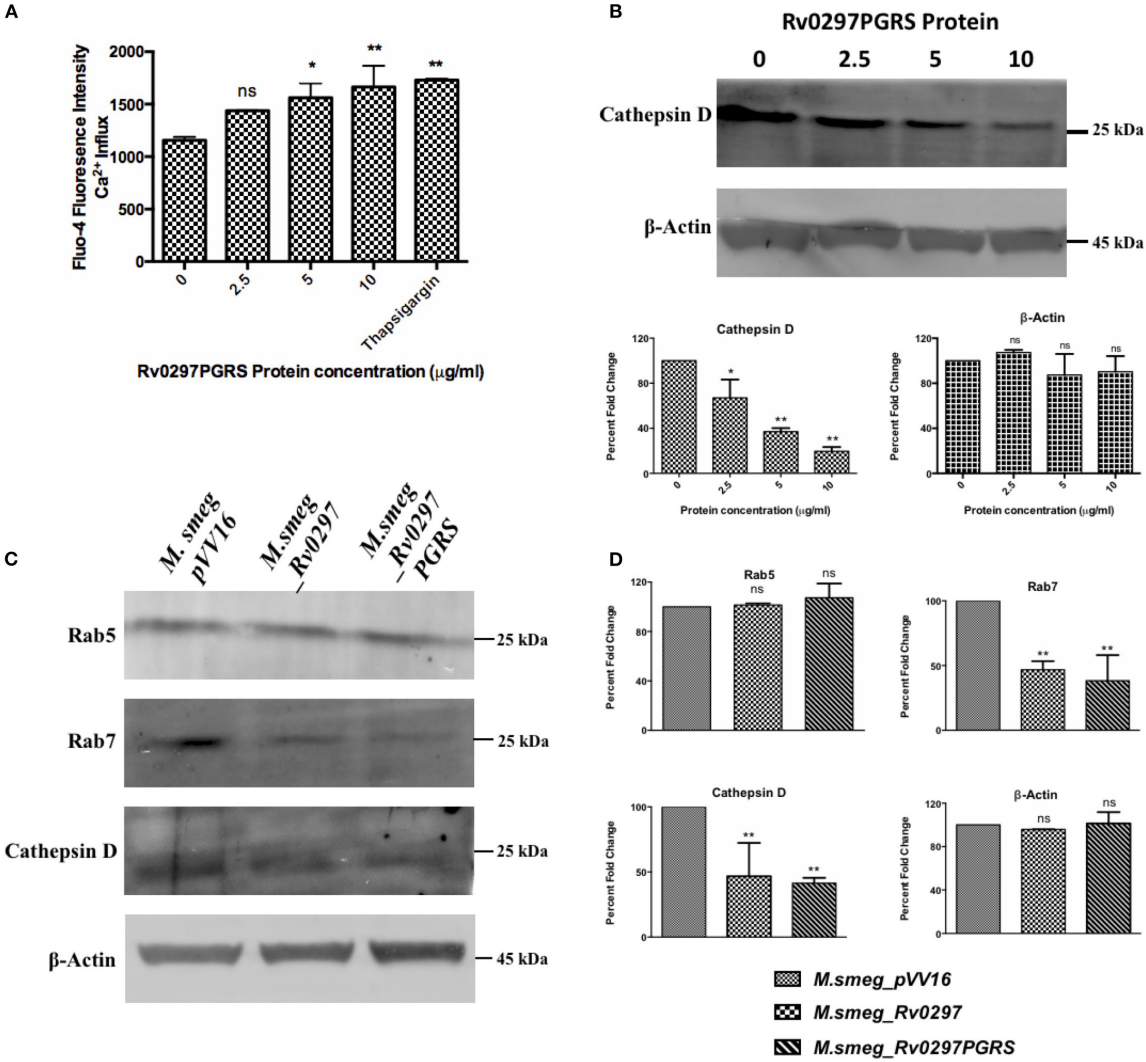
Rv0297 downregulated the phagolysosomal acidification. Ca^2+^ release from Rv0297PGRS-stimulated (for 30 h) THP-1 cells measured by Fluo-4 dye **(A)**. Thapsigargin (1 mM) was used as the positive control. **(B,C)** Western blots depicting the downregulation of the early and late phagosomal markers (Rab5, Rab7, and cathepsin D). **(B)** The levels of cathepsin D were assessed upon stimulation of the macrophages with Rv0297PGRS for 30 h. **(C)** Levels of the early and late phagosomal markers were assessed in THP-1 macrophages infected with *M.smeg_VC, M.smeg_Rv0297*, and *M.smeg_Rv0297PGRS* for 48 h. To ensure the equal loading of lysates, β-actin levels were loaded and immunoblotted. The data shown are representative of three independent experiments. **(D)** Densitometric analysis of the Western blots depicted in **(C)**. *P < 0.05, **P < 0.01, and *P* > 0.05 (ns).

Inhibition of phagolysosomal acidification and resistance against the microbicidal components of phagolysosomes are two of the important survival strategies used by *M.tb* for containment of infection (Armstrong and Hart, 1971; Vergne et al., 2003, 2004). The maturation process of phagolysosomes depends on several factors, including calcium signaling (Vergne et al., 2003; Trimble and Grinstein, 2007). A rise in the cellular calcium levels affects the phagolysosomal acidification *via* activating calcineurin phosphatase (Malik et al., 2000). *M.tb* blocks the phagolysosomal maturation by not allowing the recruitment of late endosomal markers. Early endosomal markers include transferrin receptor, EEA-1, and Rab5, which will be exchanged by the late endosomal markers such as cathepsin D and Rab7 (Rink et al., 2005; Poteryaev et al., 2010; Thi et al., 2013). The role of the PGRS domain of Rv0297 in calcium perturbations at the host cellular level and the subsequent arrest in phagolysosomal acidification has been predicted. THP-1 cells, when treated with the rRv0297PGRS protein, result in the calcium release from the THP-1 macrophages in a dose-dependent manner (Figure 1A). The levels of cathepsin D were estimated in the rRv0297PGRS protein-treated macrophages to assess its effect on phagolysosomal maturation. It was observed that rRv0297PGRS protein treatment affected the levels of the late endosomal marker cathepsin D in THP-1 cells (Figure 1B).

These findings were further explored using recombinant *M. smegmatis* expressing full-length Rv0297 and its PGRS domain. The expression of either full-length Rv0297 or its PGRS domain does not seem to affect the *in vitro* growth of the recombinant bacterium (Supplementary Figure 1). Infection of THP-1 macrophages with r*M.smeg* expressing full-length Rv0297 and the Rv0297PGRS domain has been observed to inhibit the maturation of phagolysosomes as compared to the vector control. Early and late phagolysosomal markers were assessed by the immunoblotting of lysates from r*M.smeg*-infected macrophages using anti-Rab5 (CST), anti-Rab7 (CST), and anti-cathepsin D (Cloud-Clone Corp.) antibodies. All three recombinant *M. smegmatis* expressing Rv0297, Rv0297PGRS, and the vector display normal levels of the early endosomal marker Rab5 (Figure 1C, panel 1). In contrast, probing with the late endosomal marker Rab7 showed a more than 50% reduction in the case of *M. smegmatis* expressing Rv0297 and Rv0297PGRS as compared to the vector control pVV16 (Figure 1C, panel 2). The levels of cathepsin D in the lysates of the infected macrophages were also reduced to more than 50% in the case of *M.smeg_Rv0297* and *M.smeg_Rv0297PGRS* (Figure 1C, panel 3). Both the late endosomal markers were downregulated, depicting that Rv0297 was interfering with the maturation of phagolysosomes (Figures 1C,D). Moreover, the reduction level was higher in the macrophages infected with r*M.smeg* expressing only the PGRS domain as compared to the full-length protein.

### *M.smeg_Rv0297PGRS* Leads to the Production of NO and Apoptosis of Infected Host Cells

**Figure 2:**
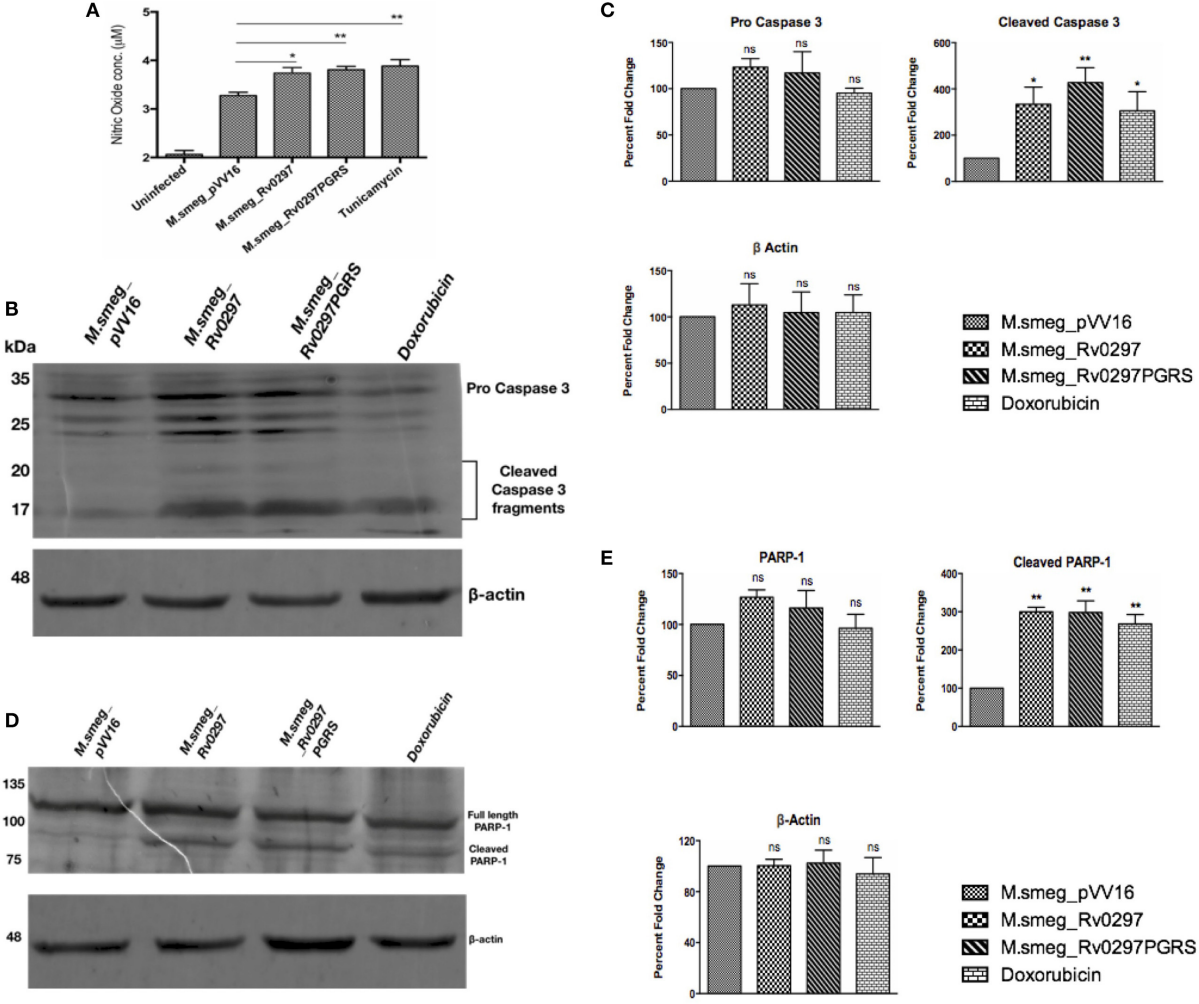
Rv0297 induces the production of nitric oxide (NO) followed by the apoptosis of host cells. **(A)** NO production by RAW264.7 macrophages upon infection with recombinant *Mycobacterium smegmatis* for 30 h. Tunicamycin (1 μm) was used as the positive control. Data were plotted as NO concentrations (in micromolars). Apoptosis was assayed by assessment of cleaved caspase 3 and poly(ADP-ribose) polymerase (PARP) in infected THP-1 macrophages. **(B,C)** Cleavage of pro-caspase 3 to active caspase 3 in THP-1 cells upon infection with recombinant *M. smegmatis* for 48 h. **(D,E)** Cleavage of full-length PARP in THP-1 cells upon infection with recombinant *M. smegmatis* for 48 h. Recombinant *M. smegmatis* with just the pVV16 vector was used as the negative control; doxorubicin was the positive control for the induction of apoptosis. Densitometric analysis of Western blots are depicted in **(C,E)**. *P < 0.05, **P < 0.01, and *P* > 0.05 (ns).

Apoptosis, a programmed cell death, generally protects the host cells by clearing the infection in the initial stages. However, it can favor the bacterium in the later stages of infection by disseminating the disease *via* apoptotic bodies. We accordingly investigated the effect of the PGRS domain of Rv0297 in macrophages infected with *rM.smeg_Rv0297* and *rM.smeg_Rv0297PGRS*. For quantification of nitric oxide release, infection was done in RAW264.7 macrophages for 30 h. For the detection of apoptosis, PMA-differentiated THP-1 cells were infected with recombinant *M. smegmatis* expressing either full-length Rv0297 or the Rv0297PGRS domain. The levels of NO were found to be upregulated in the macrophages infected with Rv0297-expressing bacteria as compared to the vector control (Figure 2A). The levels were similar in both full-length Rv0297 and its PGRS domain, reflecting the fact that the effect was solely due to the PGRS domain. Extending this result, the capability of the recombinant strains to induce apoptosis was investigated by probing against apoptotic markers. It was observed that Rv0297 and its PGRS domain were efficiently able to induce apoptosis of the infected macrophages after 48 h, as evident from the cleavage of caspase 3 to activated caspase 3 fragments (Figures 2B,C). Similar effects were observed in the case of poly(ADP-ribose) polymerase (PARP) cleavage as a marker of apoptosis (Figures 2D,E). These results indicate that the Rv0297PGRS domain provides the capability to non-pathogenic bacterium to stimulate NO production from host macrophages, followed by macrophage cell death by apoptosis. In contrast, *M.smeg_*pVV16 (the vector control), being devoid of Rv0297, is incapable of inducing such responses in macrophages (Figure 2).

### Rv0297PGRS Domain Confers Resistance to Oxidative and Hypoxic Stress Conditions

**Figure 3:**
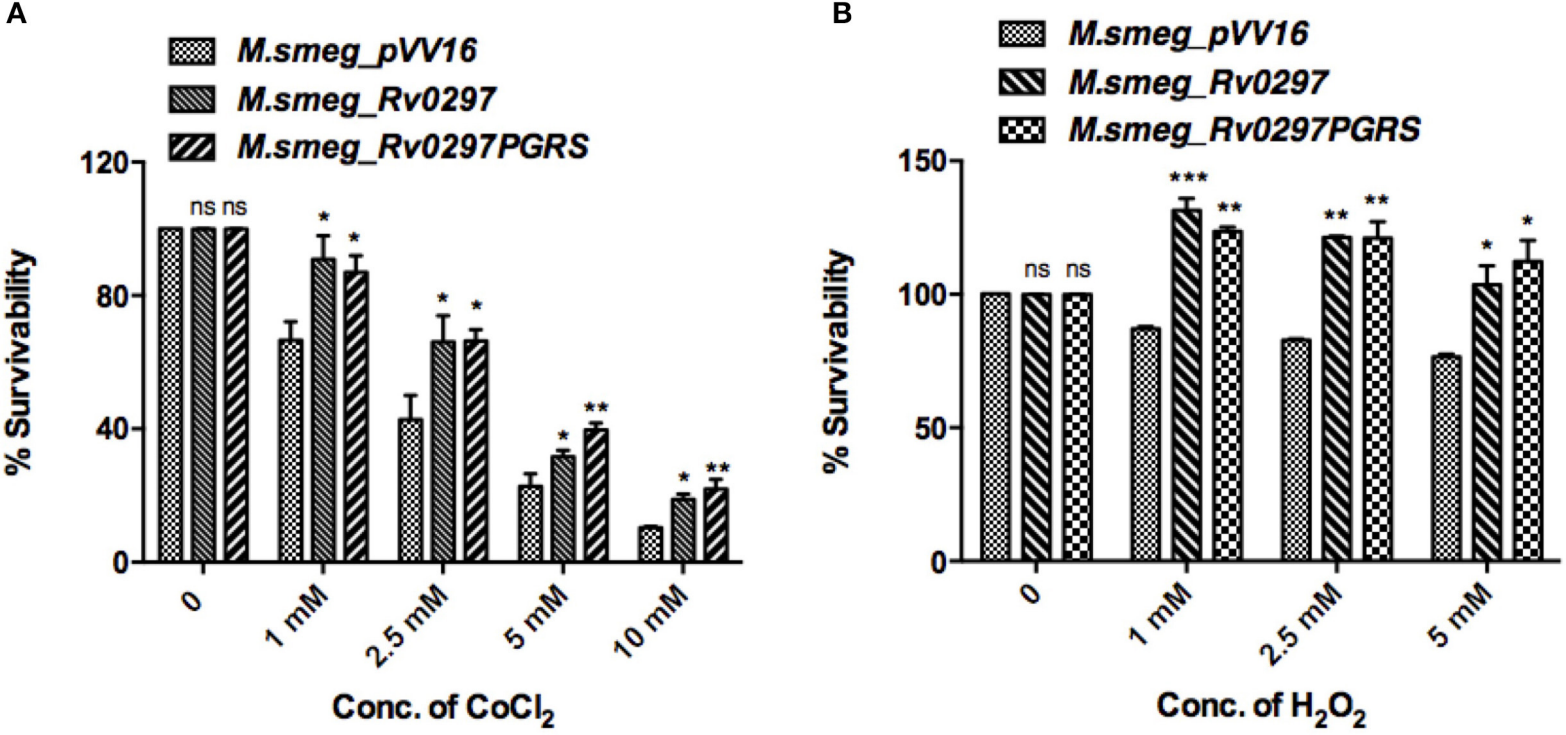
*Mycobacterium tuberculosis* Rv0297PGRS protects the bacterium against oxidative and hypoxic stress conditions. Recombinant *M.smeg_pVV16, M.smeg_Rv0297*, and *M.smeg_Rv0297PGRS* bacterial cells were grown in the presence of hypoxic (CoCl_2_) **(A)** and oxidative (H_2_O_2_) **(B)** stress environments. Cell viability was assessed using 0.3% Resazurin sodium salt for 4 h spectrophotometrically. Data were plotted as percent survivability. 26. All values were represented as mean+SD from three independent experminets. *P < 0.05, **P < 0.01, ***P < 0.001, and *P* > 0.05 (ns).

Apoptotic bodies disseminate bacteria to nearby cells, thus leading to the progression of infection. Infected macrophages present a microbicidal environment in the form of acidic and hypoxic stresses to degrade the mycobacteria. *M.tb* presents an alternative survival strategy for residence in macrophages by attaining resistance against acidic and hypoxic stress environments (Gomes et al., 1999; Vandal et al., 2009). Mycobacterium secretes several proteins, which provide it protection against oxidative burst and hypoxic conditions (Stewart et al., 2005; Singh et al., 2016). H_2_O_2_ and CoCl_2_ were reported to create oxidative and hypoxic stress conditions, respectively (Piret et al., 2002; Wijeratne et al., 2005; Singh et al., 2016). Survival of r*M.smeg* expressing full-length Rv0297 as well as only the PGRS domain was examined using Resazurin sodium salt. Recombinant *M. smegmatis* that expresses Rv0297 and Rv0297PGRS presented a higher survival percentage in the presence of hypoxic conditions of 1–10mM of CoCl_2_ as compared to the *M. smegmatis* expressing the vector control pVV16 (Figure 3A). The vector control *M.smeg_pVV16* was also unable to grow beyond the 1 mM concentration of H_2_O_2_. In contrast, bacteria expressing either Rv0297 or Rv0297PGRS protein were growing well even in the presence of 5 mM H_2_O_2_ (Figure 3B). These results show that *M.tb* full-length Rv0297 or the PGRS domain alone may play a role in resisting macrophage stress conditions.

### Rv0297 Enhances Bacterial Survival in Macrophages

**Figure 4:**
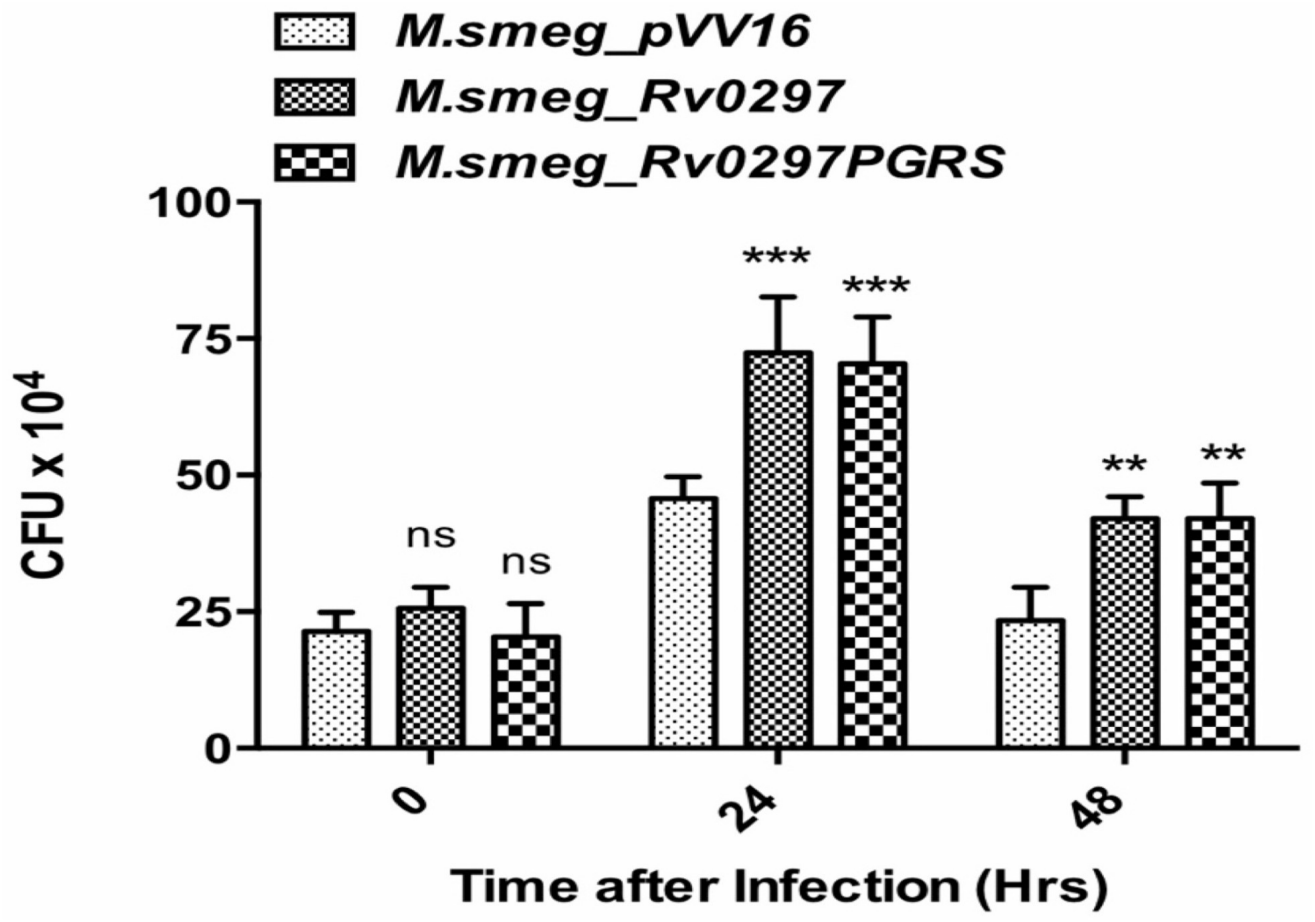
Rv0297 provides survival advantage to *Mycobacterium smegmatis* in infected macrophages. THP-1 macrophages were infected with *M. smegmatis* expressing Rv0297 full-length protein or the PGRS domain only, and bacterial load was assessed 24 or 48 h post-infection. All values were represented as the mean ± SD from three independent experiments. **P < 0.01, ***P < 0.001, and *P* > 0.05 (ns).

Extending the above results, we explored the bacillary persistence in infected macrophages. To further investigate the role of Rv0297PGRS in enhancing the survival ability of *M. smegmatis* inside THP-1 macrophages, a CFU assay was performed and the intracellular bacillary survival of r*M.smeg_pVV16*, r*M.smeg_Rv0297*, and r*M.smeg_Rv0297PGRS* was compared. PMA-differentiated THP-1 macrophages were infected with recombinant *M. smegmatis* constructs at an MOI of 1:10 at 37°C for 3 h, followed by washing and gentamicin treatment to remove extracellular bacteria. The intracellular growth of bacteria was assayed by enumerating the CFU at different time points post-infection. r*M.smeg_Rv0297* and r*M.smeg_Rv0297PGRS* were found to be surviving better than r*M.smeg_pVV16* during the course of infection in THP-1 macrophages (Figure 4). This clearly shows that recombinant *M. smegmatis* expressing the Rv0297 protein either full length or only the PGRS domain displays enhanced survival in human macrophage cell lines, signifying a probable role of Rv0297 in bacterial persistence. No significant difference in response of either full length or its PGRS domain was observed, thereby implying that the PGRS domain alone was significant (Figure 4).

### Rv0297PGRS Is Also Involved in the Modulation of Immune Responses

The PE/PPE/PE_PGRS proteins of *M.tb* have very high antigenic indexes and are able to evoke immune responses to modulate the host responses upon *M.tb* infection (Choudhary et al., 2003; Chakhaiyar et al., 2004; Singh et al., 2008; Tundup et al., 2008; Nair et al., 2009; Bansal et al., 2010; Cohen et al., 2014). The antigenic index of Rv0297 is 1.98 as compared to the other well-studied proteins—PE_PGRS33, PE_PGRS62, and PE_PGRS17 (antigenic indices of 1.6981, 0.3496, and 0.8921, respectively)—using the VaxiJen Antigenecity Prediction tool. Memory T cells against Rv0297 have been shown to be present in latently *M.tb*-infected individuals (Lindestam Arlehamn et al., 2013).

**Figure 5:**
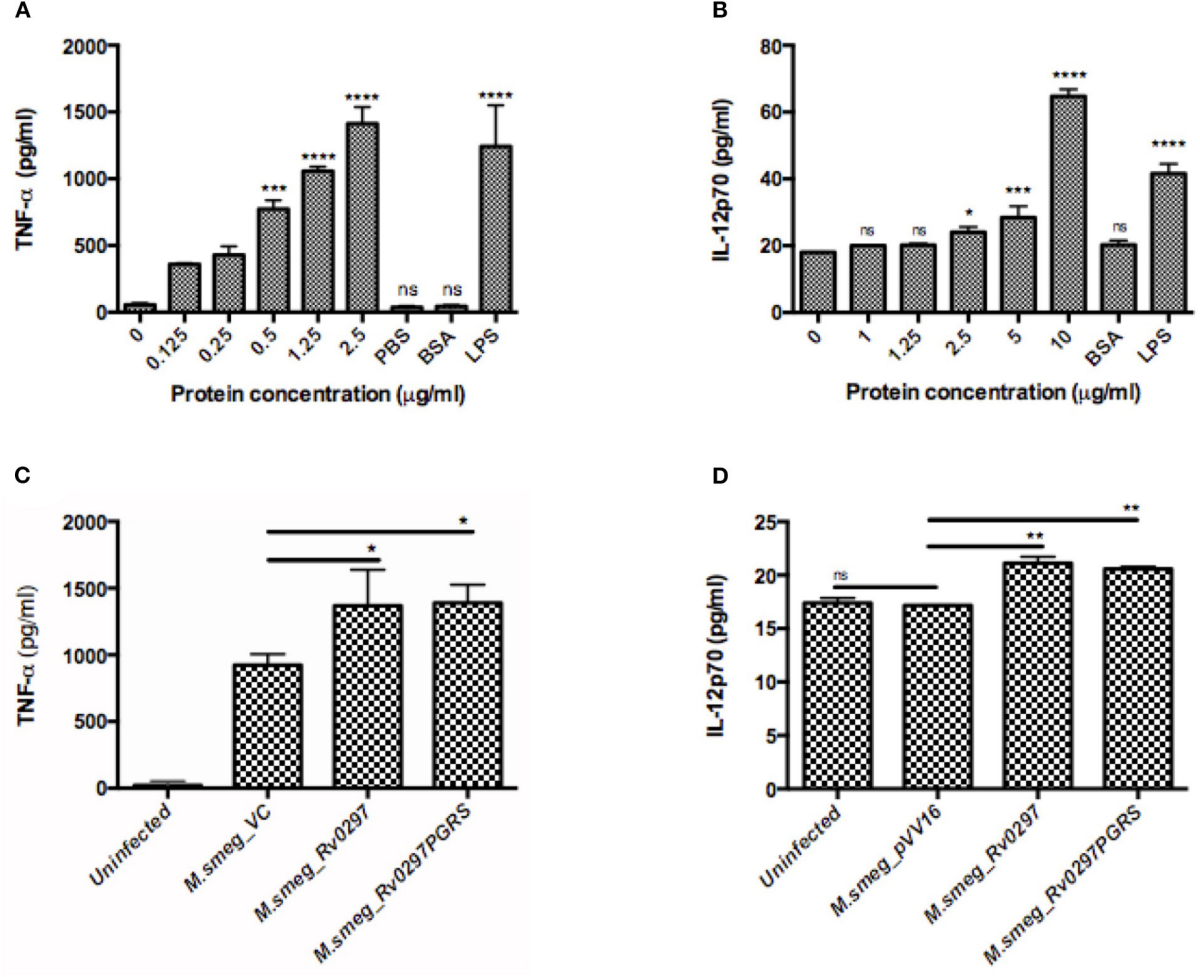
Rv0297PGRS is involved in the immunomodulation of host macrophages. THP-1 cells were either treated with different concentrations of Rv0297 protein or infected with recombinant *Mycobacterium smegmatis* expressing Rv0297PGRS, and production of TNF-α **(A,B)** and IL-12p70 **(C,D)** cytokines was assessed by ELISA (eBiosciences) as per the manufacturer's instruction. All values were represented as the mean ± SD from three independent experiments. *P < 0.05, **P < 0.01, ***P < 0.001, ****P < 0.0001, and *P* > 0.05 (ns).

To investigate the likely role of Rv0297PGRS in the modulation of host immune responses, THP-1 cells were either treated with different concentrations of Rv0297PGRS protein or infected with r*M.smeg* expressing Rv0297PGRS, followed by assessment of TNF-α and IL-12p70 cytokine production by ELISA. In both the cases, Rv0297 was found to stimulate the production of higher levels of TNF-α, which is a pro-inflammatory cytokine (Figures 5A,B). With the increased concentrations of proteins, the levels of IL-12p70 (Figure 5C) also increased, thus pointing toward the immunomodulatory role of Rv0297. Non-immunogenic protein BSA (10 μg/ml) does not lead to any cytokine production. In comparison to that, 200 ng/ml of LPS induced significant amounts of both cytokines, as predicted. The production of TNF-α and IL-12p70 from macrophages was correspondingly higher when the cells were infected with *M.smeg_Rv0297* and *M.smeg_Rv0297PGRS* as compared to the vector control and uninfected (Figures 5B,D). A major cytokine involved in lung granuloma formation is TNF-α (Tufariello et al., 2003). In contrast, the production of Rv0297 in the later stages of infection and the subsequent induction of TNF-α release may aid in granuloma maintenance. The immune response thus generated may be linked to the activation of chemokine essential for the recruitment of macrophages and the maintenance of lung granulomas.

## Discussion

*M.tb* secretes several proteins which modulate the cellular cascades, such as the production of ROS and NO intermediates (Stamm et al., 2015), host cell apoptosis (Harding and Boom, 2010), antigen presentation (Mahajan et al., 2012), and phagosomal acidification (Kleinnijenhuis et al., 2011), in its favor for initiating disease pathogenesis. The unique presence of PE_PGRS proteins in the pathogenic strains of *Mycobacterium* is an indicator of their likely role in the pathogenesis and virulence of tuberculosis (Ramakrishnan et al., 2000; Brennan and Delogu, 2002; Delogu et al., 2004; Akhter et al., 2012). The high sequence variations and repetitive nature of PE/PPE/PE_PGRS proteins have been implicated in antigenic variation and immune evasion (Cole et al., 1998; Banu et al., 2002; Talarico et al., 2005; Akhter et al., 2012). The PE_PGRS family of proteins are majorly secreted or surface-exposed and play crucial roles in host–pathogen interactions (Banu et al., 2002; Delogu et al., 2004). In our previous study, the Rv0297PGRS domain was reported to localize to the endoplasmic reticulum (ER) of host cells and thereby evoke an ER stress-mediated stress response. The stress-generated response was dependent on the interactions between Rv0297PGRS and TLR4 (Grover et al., 2018).

*M.tb*, an intracellular pathogen, survives within host macrophages *via* a number of intricate mechanisms. One of the important survival strategies of pathogenic *M.tb* is their survival inside acidified macrophages and inhibition of the acidification of phagosomes (Armstrong and Hart, 1971; Vergne et al., 2003, 2004). Involvement of calcium signaling is the critical feature of this process (Malik et al., 2001, 2003). PE_PGRS proteins, having calcium-binding domains, were thought to bind calcium ions from host cells (Bachhawat and Singh, 2007; Yeruva et al., 2016). Once *M.tb* resides in macrophages, it produces these proteins that will serve as possible calcium binders and thus disturb the physiological levels of cellular calcium, thereby inducing calcium release from ER stores (Meena, 2019). The first step of the interaction of *M.tb* with macrophages leads to altered levels of calcium signaling cascades, thereby modulating phagosomal acidification. Earlier studies have demonstrated the downregulation of this pathway by *M. tb via* inhibition of sphingosine kinase. Sphingosine kinase is a macrophage enzyme that is required for upregulated levels of cytosolic calcium (Malik et al., 2003; Kusner, 2005). All these aspects show that cytosolic calcium levels gets depleted upon *M.tb* infection in human macrophages, which thereby alters the maturation of phagolysosomes; this is used as their survival strategy (Malik et al., 2000; Stober et al., 2001).

The *M. bovis* BCG mutant lacking PE_PGRS5 has been found to be enriched in acidified phagosomes in a transposon mutant library screening, indicating a possible function of this protein in arresting the acidification of phagolysosomes (Stewart et al., 2005). Thus, its function in phagosomal acidification during TB pathogenesis was investigated in the present study. Macrophages infected with *M. smegmatis* expressing full-length Rv0297 and/or the PGRS domain alone have shown downregulated levels of the late endosomal markers Rab7 and cathepsin D as compared to the vector control. However, the early endosomal markers were found to be similar in *M.smeg_Rv0297, M.smeg_Rv0297PGRS*, and *M.smeg_pVV16*. These results show that the Rv0297PGRS domain was able to affect the process of phagosomal acidification by downregulating the levels of macrophage late endosomal markers such as Rab7 and cathepsin D. The explicit role of PE-PGRS11 and PE_PGRS30 in mediating mycobacterial resistance to oxidative stress (Chaturvedi et al., 2010) and inhibition of phagolysosomal fusion (Iantomasi et al., 2012), respectively, was evident. PE_PGRS62 has been described to arrest phagosomal maturation by hindering the recruitment of Rab7 and blocking iNOS expression (Thi et al., 2013). It can be noted that these proteins, as possible calcium modulators, may affect the fusion of phagosomes with lysosomes to inhibit clearing of the mycobacterium. In addition to these observations, Rv0297-expressing *M. smegmatis* initiates the apoptotic pathway of macrophages, thus aiding in its dissemination given its expression at the later stages of infection (Kruh et al., 2010). *M.smeg_Rv0297* was found to be viable under macrophage acidic stress conditions and continued to multiply. Apoptosis has been designated to favor mycobacterial survival in the later stages. The PGRS domain of Rv0297 is also involved in evoking immune responses in terms of TNF-α and IL-12 production. TNF-α is one of the major cytokines involved in lung granuloma formation during the later stages of infection (Tufariello et al., 2003). As Rv0297 was found to be expressed at the later stages of tuberculosis infection (Kruh et al., 2010), the immune response thus generated may be linked to the recruitment of macrophages and maintenance of lung granulomas. All these observations point to hitherto unknown roles of Rv0297 in modulating macrophage functions along with providing protection to the infected bacterium.

In our previous study, the ER stress-mediated apoptosis of Rv0297PGRS was found to be TLR4-dependent. Moreover, the virulence and pathogenesis of *M.tb* depends on the interaction of mycobacterial ligands with TLRs, including TLR1, TLR2, TLR4, and TLR9, along with their associated signaling cascades, such as apoptosis and immune system activation (Means et al., 1999, 2001; Tapping and Tobias, 2003; Bulut et al., 2005). Diverse mycobacterial components activate the formation of TLR heterodimers (TLR1 with TLR2 and TLR4 with TLR6) to initiate downstream signaling cascades (Drennan et al., 2004; Krutzik and Modlin, 2004). The modulation and attenuation of immune responses during *M.tb* infection in RAW264.7 and THP-1 cells have been shown to be dependent on TLR4 signaling-mediated upregulation of the host microRNA (Niu et al., 2018). Thus, there is a need to unveil the specific roles of TLR functions using knockdown studies of multiple TLRs (Bafica et al., 2005). The dependency of TLRs in evoking immune responses by Rv0297PGRS can be further investigated to unravel the involvement of a possible crosstalk mechanism between different TLRs.

In conclusion, our results demonstrate the novel functions employed by *M.tb* through the PGRS domain of Rv0297. Rv0297PGRS is able to induce calcium release from stimulated cells, as evidenced by the Fluo-4 calcium release assay. Subsequently, Rv0297 was observed to interfere with the phagosomal acidification process by downregulating the expression of the late endosomal markers Rab7 and cathepsin D in infected macrophages. Rv0297PGRS has been found to induce the apoptosis of infected cells for bacterial survival and thereby aids in the dissemination of the infection to nearby cells. The PGRS domain of Rv0297 also enhances the survivability of recombinant bacterium under the highly acidic environment of the macrophages. Hence, Rv0297-encoded PE_PGRS5 may have a role in the calcium-modulated host responses during *M.tb* infection *via* altering macrophage functions.

## Data Availability Statement

All datasets presented in this study are included in the article/Supplementary Material.

## Author Contributions

SH, NE and SG conceptualized and designed the research. TS performed the experiments. TS, NA, SG, and MP carried out data analysis. TS, NE, and SH wrote the manuscript. All authors approved the final version.

## Conflict of Interest

The authors declare that the research was conducted in the absence of any commercial or financial relationships that could be construed as a potential conflict of interest.

## Supplementary Material

Supplementary Figure 1 Growth curve of recombinant *M. smegmatis* expressing full length Rv0297 or its PGRS domain. Growth curve analysis showed that expression of Rv0297 is not affceting the *in vitro* growth of recombinant bacterium.

## References

[B1] Aguiló N. Marinova D. Martín C. Pardo J. (2013). ESX-1-induced apoptosis during mycobacterial infection: to be or not to be, that is the question. Front. Cell. Infect. Microbiol. 3:88. 10.3389/fcimb.2013.00088 24364000 PMC3850411

[B2] Ahmad J. Farhana A. Pancsa R. Arora S. K. Srinivasan A. Tyagi A. K.. (2018). Contrasting function of structured N-terminal and unstructured C-terminal segments of *Mycobacterium tuberculosis* PPE37 protein. MBio. 9:e01712–17. 10.1128/mBio.01712-17 29362230 PMC5784249

[B3] Ahmad J. Khubaib M. Sheikh J. A. Pancsa R. Kumar S. Srinivasan A.. (2020). Disorder-to-order transition in PE–PPE proteins of *Mycobacterium tuberculosis* augments the pro-pathogen immune response. FEBS Open Bio. 10, 70–85. 10.1002/2211-5463.12749 31643141 PMC6943233

[B4] Akhter Y. Ehebauer M. Mukhopadhyay S. Hasnain S. (2012). The PE/PPE multigene family codes for virulence factors and is a possible source of mycobacterial antigenic variation: perhaps more? Biochimie. 94, 110–116. 10.1016/j.biochi.2011.09.026 22005451

[B5] Armstrong J. A. Hart P. D. A. (1971). Response of cultured macrophages to *Mycobacterium tuberculosis* with observations on fusion of lysosomes with phagosomes. J. Exp. Med. 134:713. 10.1084/jem.134.3.713 15776571 PMC2139093

[B6] Bachhawat N. Singh B. (2007). Mycobacterial PE_PGRS proteins contain calcium-binding motifs with parallel beta-roll folds. Genomics Proteomics Bioinform. 5, 236–241. 10.1016/S1672-0229(08)60010-8 18267304 PMC5054227

[B7] Bafica A. Scanga C. A. Feng C. G. Leifer C. Cheever A. Sher A. (2005). TLR9 regulates Th1 responses and cooperates with TLR2 in mediating optimal resistance to *Mycobacterium tuberculosis*. J. Exp. Med. 202, 1715–1724. 10.1084/jem.20051782 16365150 PMC2212963

[B8] Bansal K. Elluru S. Narayana Y. Chaturvedi R. Patil S. Kaveri S.. (2010). PE_PGRS antigens of *Mycobacterium tuberculosis* induce maturation and activation of human dendritic cells. J. Immunol. 184, 3495–3504. 10.4049/jimmunol.0903299 20176745

[B9] Banu S. Honor,é N. Saint-Joanis B. Philpott D. Prévost M.-C. Cole S. T. (2002). Are the PE-PGRS proteins of *Mycobacterium tuberculosis* variable surface antigens? Mol. Microbiol. 44, 9–19. 10.1046/j.1365-2958.2002.02813.x 11967065

[B10] Basu S. Pathak S. K. Banerjee A. Pathak S. Bhattacharyya A. Yang Z.. (2007). Execution of macrophage apoptosis by PE_PGRS33 of *Mycobacterium tuberculosis* is mediated by Toll-like receptor 2-dependent release of tumor necrosis factor-alpha. J. Biol. Chem. 282, 1039–1050. 10.1074/jbc.M604379200 17095513

[B11] Becq J. Gutierrez M. C. Rosas-Magallanes V. Rauzier J. Gicquel B. Neyrolles O.. (2007). Contribution of horizontally acquired genomic islands to the evolution of the tubercle bacilli. Mol. Biol. Evol. 24, 1861–1871. 10.1093/molbev/msm111 17545187

[B12] Brennan M. J. Delogu G. (2002). The PE multigene family: a 'molecular mantra' for mycobacteria. Trends Microbiol. 10, 246–249. 10.1016/S0966-842X(02)02335-1 11973159

[B13] Bulut Y. Michelsen K. Hayrapetian L. Naiki Y. Spallek R. Singh M.. (2005). *Mycobacterium tuberculosis* heat shock proteins use diverse toll-like receptor pathways to activate pro-inflammatory signals. J. Biol. Chem. 280, 20961–20967. 10.1074/jbc.M411379200 15809303

[B14] Chaitra M. G. Shaila M. S. Nayak R. (2007). Evaluation of T-cell responses to peptides with MHC class I-binding motifs derived from PE_PGRS 33 protein of *Mycobacterium tuberculosis*. J. Med. Microbiol. 56, 466–474. 10.1099/jmm.0.46928-0 17374885

[B15] Chakhaiyar P. Nagalakshmi Y. Aruna B. Murthy K. J. Katoch V. M. Hasnain S. E. (2004). Regions of high antigenicity within the hypothetical PPE major polymorphic tandem repeat open-reading frame, Rv2608, show a differential humoral response and a low T cell response in various categories of patients with tuberculosis. J. Infect. Dis. 190, 1237–1244. 10.1086/423938 15346333

[B16] Chaturvedi R. Bansal K. Narayana Y. Kapoor N. Sukumar N. Togarsimalemath S. K.. (2010). The multifunctional PE_PGRS11 protein from *Mycobacterium tuberculosis* plays a role in regulating resistance to oxidative stress. J. Biol. Chem. 285, 30389–30403. 10.1074/jbc.M110.135251 20558725 PMC2945531

[B17] Choudhary R. K. Mukhopadhyay S. Chakhaiyar P. Sharma N. Murthy K. J. Katoch V. M.. (2003). PPE antigen Rv2430c of *Mycobacterium tuberculosis* induces a strong B-cell response. Infect. Immun. 71, 6338–6343. 10.1128/IAI.71.11.6338-6343.2003 14573653 PMC219563

[B18] Clapham D. E. (2007). Calcium signaling. Cell 131, 1047–1058. 10.1016/j.cell.2007.11.028 18083096

[B19] Cohen I. Parada C. Acosta-Gío E. Espitia C. (2014). The PGRS domain from PE_PGRS33 of *Mycobacterium tuberculosis* is target of humoral immune response in mice and humans. Front. Immunol. 5:23. 10.3389/fimmu.2014.00236 24904584 PMC4033847

[B20] Cole S. T. Brosch R. Parkhill J. Garnier T. Churcher C. Harris D.. (1998). Deciphering the biology of *Mycobacterium tuberculosis* from the complete genome sequence (vol 393, pg 537, 1998). Nature. 396, 190–198. 10.1038/24206 9634230

[B21] Delogu G. Pusceddu C. Bua A. Fadda G. Brennan M. J. Zanetti S. (2004). Rv1818c-encoded PE_PGRS protein of *Mycobacterium tuberculosis* is surface exposed and influences bacterial cell structure. Mol. Microbiol. 52, 725–733. 10.1111/j.1365-2958.2004.04007.x 15101979

[B22] Drennan M. B. Nicolle D. Quesniaux V. J. F. Jacobs M. Allie N. Mpagi J.. (2004). Toll-like receptor 2-deficient mice succumb to *Mycobacterium tuberculosis* infection. Am. J. Pathol. 164, 49–57. 10.1016/S0002-9440(10)63095-7 14695318 PMC1602241

[B23] Gomes M. S. Paul S. Moreira A. L. Appelberg R. Rabinovitch M. Kaplan G. (1999). Survival of &lt;em&gt;Mycobacterium avium&lt;/em&gt; and&lt;em&gt;*Mycobacterium tuberculosis*&lt;/em&gt; in acidified vacuoles of murine macrophages. Infect. Immun. 67:3199. 10.1128/IAI.67.7.3199-3206.1999 10377091 PMC116496

[B24] Grover S. Sharma T. Singh Y. Kohli S. Manjunath P. Singh A.. (2018). The PGRS domain of *Mycobacterium tuberculosis* PE_PGRS protein Rv0297 is involved in endoplasmic reticulum stress-mediated apoptosis through toll-like receptor 4. MBio. 9. 10.1128/mBio.01017-18 29921671 PMC6016250

[B25] Harding C. V. Boom W. H. (2010). Regulation of antigen presentation by *Mycobacterium tuberculosis*: a role for Toll-like receptors. Nat. Rev. Microbiol. 8, 296–307. 10.1038/nrmicro2321 20234378 PMC3037727

[B26] Iantomasi R. Sali M. Cascioferro A. Palucci I. Zumbo A. Soldini S.. (2012). PE_PGRS30 is required for the full virulence of *Mycobacterium tuberculosis*. Cell. Microbiol. 14, 356–367. 10.1111/j.1462-5822.2011.01721.x 22050772

[B27] Khubaib M. Sheikh J. A. Pandey S. Srikanth B. Bhuwan M. Khan N.. (2016). *Mycobacterium tuberculosis* co-operonic PE32/PPE65 proteins alter host Immune responses by hampering Th1 response. Front. Microbiol. 7:719. 10.3389/fmicb.2016.00719 27242739 PMC4868851

[B28] Kleinnijenhuis J. Oosting M. Joosten L. A. B. Netea M. G. Van Crevel R. (2011). Innate immune recognition of *Mycobacterium tuberculosis*. Clin. Dev. Immunol. 2011:12. 10.1155/2011/405310 21603213 PMC3095423

[B29] Kohli S. Singh Y. Sharma K. Mittal A. Ehtesham N. Z. Hasnain S. E. (2012). Comparative genomic and proteomic analyses of PE/PPE multigene family of *Mycobacterium tuberculosis* H(3)(7)Rv and H(3)(7)Ra reveal novel and interesting differences with implications in virulence. Nucleic Acids Res. 40, 7113–7122. 10.1093/nar/gks465 22618876 PMC3424577

[B30] Kruh N. A. Troudt J. Izzo A. Prenni J. Dobos K. M. (2010). Portrait of a pathogen: the *Mycobacterium tuberculosis* proteome in vivo. PLoS ONE. 5:e13938. 10.1371/journal.pone.0013938 21085642 PMC2978697

[B31] Krutzik S. R. Modlin R. L. (2004). The role of Toll-like receptors in combating mycobacteria. Semin. Immunol. 16, 35–41. 10.1016/j.smim.2003.10.005 14751762

[B32] Kusner D. J. (2005). Mechanisms of mycobacterial persistence in tuberculosis. Clin. Immunol. 114:239–247. 10.1016/j.clim.2004.07.016 15721834

[B33] Lindestam Arlehamn C. S. Gerasimova A. Mele F. Henderson R. Swann J. Greenbaum J. A.. (2013). Memory T cells in latent *Mycobacterium tuberculosis* infection are directed against three antigenic islands and largely contained in a CXCR3+CCR6+ Th1 subset. PLoS Pathog. 9:e1003130. 10.1371/journal.ppat.1003130 23358848 PMC3554618

[B34] Mahajan S. Dkhar H. K. Chandra V. Dave S. Nanduri R. Janmeja A. K.. (2012). *Mycobacterium tuberculosis* modulates macrophage lipid-sensing nuclear receptors PPARγ and TR4 for survival. J. Immunol. 188, 5593–5603. 10.4049/jimmunol.1103038 22544925

[B35] Malik Z. A. Denning G. M. Kusner D. J. (2000). Inhibition of Ca(2+) signaling by *Mycobacterium tuberculosis* is associated with reduced phagosome-lysosome fusion and increased survival within human macrophages. J. Exp. Med. 191, 287–302. 10.1084/jem.191.2.287 10637273 PMC2195750

[B36] Malik Z. A. Iyer S. S. Kusner D. J. (2001). *Mycobacterium tuberculosis* phagosomes exhibit altered calmodulin-dependent signal transduction: contribution to inhibition of phagosome-lysosome fusion and intracellular survival in human macrophages. J. Immunol. 166, 3392–3401. 10.4049/jimmunol.166.5.3392 11207296

[B37] Malik Z. A. Thompson C. R. Hashimi S. Porter B. Iyer S. S. Kusner D. J. (2003). Cutting edge: *Mycobacterium tuberculosis* blocks Ca2+ signaling and phagosome maturation in human macrophages via specific inhibition of sphingosine kinase. J. Immunol. 170, 2811–2815. 10.4049/jimmunol.170.6.2811 12626530

[B38] Means T. K. Jones B. Schromm A. B. Shurtleff B. A. Smith J. A. Keane J.. (2001). Differential effects of a toll-like receptor antagonist on *Mycobacterium tuberculosis*-induced macrophage responses. J. Immunol. 166, 4074–4082. 10.4049/jimmunol.166.6.4074 11238656

[B39] Means T. K. Wang S. Lien E. Yoshimura A. Golenbock D. T. Fenton M. J. (1999). Human toll-like receptors mediate cellular activation by *Mycobacterium tuberculosis*. J. Immunol. 163:3920. 10490993

[B40] Meena L. S. (2019). Interrelation of Ca2+ and PE_PGRS proteins during *Mycobacterium tuberculosis* pathogenesis. J. Biosci. 44:24. 10.1007/s12038-018-9828-4 30837375

[B41] Nair S. Ramaswamy P. A. Ghosh S. Joshi D. C. Pathak N. Siddiqui I.. (2009). The PPE18 of *Mycobacterium tuberculosis* interacts with TLR2 and activates IL-10 induction in macrophage. J. Immunol. 183, 6269–6281. 10.4049/jimmunol.0901367 19880448

[B42] Niu W. Sun B. Li M. Cui J. Huang J. Zhang L. (2018). TLR-4/microRNA-125a/NF-κB signaling modulates the immune response to *Mycobacterium tuberculosis* infection. Cell Cycle (Georgetown, Tex.). 17, 1931–1945. 10.1080/15384101.2018.1509636 30153074 PMC6152532

[B43] Piret J.-P. Mottet D. Raes M. Michiels C. (2002). CoCl2, a chemical inducer of hypoxia-inducible factor-1, and hypoxia reduce apoptotic cell death in hepatoma cell line HepG2. Ann. N. Y. Acad. Sci. 973, 443–447. 10.1111/j.1749-6632.2002.tb04680.x 12485908

[B44] Poteryaev D. Datta S. Ackema K. Zerial M. Spang A. (2010). Identification of the switch in early-to-late endosome transition. Cell. 141, 497–508. 10.1016/j.cell.2010.03.011 20434987

[B45] Ramakrishnan L. Federspiel N. A. Falkow S. (2000). Granuloma-specific expression of Mycobacterium virulence proteins from the glycine-rich PE-PGRS family. Science 288, 1436–1439. 10.1126/science.288.5470.1436 10827956

[B46] Rink J. Ghigo E. Kalaidzidis Y. Zerial M. (2005). Rab conversion as a mechanism of progression from early to late endosomes. Cell. 122, 735–749. 10.1016/j.cell.2005.06.043 16143105

[B47] Ruckdeschel K. Roggenkamp A. Lafont V. Mangeat P. Heesemann J. Rouot B. (1997). Interaction of *Yersinia enterocolitica* with macrophages leads to macrophage cell death through apoptosis. Infect. Immun. 65, 4813–4821 10.1128/IAI.65.11.4813-4821.1997 9353070 PMC175691

[B48] Singh P. Rao R. Reddy J. Prasad R. B. N. Kotturu S. K. Ghosh S.. (2016). PE11, a PE/PPE family protein of *Mycobacterium tuberculosis* is involved in cell wall remodeling and virulence. Sci. Rep. 6:21624. 10.1038/srep21624 26902658 PMC4763214

[B49] Singh P. P. Parra M. Cadieux N. Brennan M. J. (2008). A comparative study of host response to three *Mycobacterium tuberculosis* PE_PGRS proteins. Microbiology (Reading,. Engl). 154, 3469–3479. 10.1099/mic.0.2008/019968-0 18957600

[B50] Stamm C. E. Collins A. C. Shiloh M. U. (2015). Sensing of *Mycobacterium tuberculosis* and consequences to both host and bacillus. Immunol. Rev. 264, 204–219. 10.1111/imr.12263 25703561 PMC4339209

[B51] Stewart G. R. Patel J. Robertson B. D. Rae A. Young D. B. (2005). Mycobacterial mutants with defective control of phagosomal acidification. PLoS Pathog. 1, 269–278. 10.1371/journal.ppat.0010033 16322769 PMC1291353

[B52] Stober C. B. Lammas D. A. Li C. M. Kumararatne D. S. Lightman S. L. McArdle C. A. (2001). ATP-mediated killing of *Mycobacterium bovis* bacille Calmette-Guerin within human macrophages is calcium dependent and associated with the acidification of mycobacteria-containing phagosomes. J. Immunol. 166, 6276–6286. 10.4049/jimmunol.166.10.6276 11342651

[B53] Talarico S. Cave M. D. Marrs C. F. Foxman B. Zhang L. Yang Z. (2005). Variation of the &lt;em&gt;*Mycobacterium tuberculosis*&lt;/em&gt; PE_PGRS33 gene among clinical isolates. J. Clin. Microbiol. 43:4954. 10.1128/JCM.43.10.4954-4960.2005 16207947 PMC1248487

[B54] Tapping R. I. Tobias P. S. (2003). Mycobacterial lipoarabinomannan mediates physical interactions between TLR1 and TLR2 to induce signaling. J. Endotoxin Res. 9, 264–268. 10.1177/09680519030090040801 12935358

[B55] Thi E. P. Hong C. J. Sanghera G. Reiner N. E. (2013). Identification of the *Mycobacterium tuberculosis* protein PE-PGRS62 as a novel effector that functions to block phagosome maturation and inhibit iNOS expression. Cell. Microbiol. 15, 795–808. 10.1111/cmi.12073 23167250

[B56] Tiwari B. Soory A. Raghunand T. R. (2014). An immunomodulatory role for the *Mycobacterium tuberculosis* region of difference 1 locus proteins PE35 (Rv3872) and PPE68 (Rv3873). FEBS J. 281, 1556–1570. 10.1111/febs.12723 24467650

[B57] Tiwari B. M. Kannan N. Vemu L. Raghunand T. R. (2012). The *Mycobacterium tuberculosis* PE proteins Rv0285 and Rv1386 modulate innate immunity and mediate bacillary survival in macrophages. PLoS ONE. 7:e51686. 10.1371/journal.pone.0051686 23284742 PMC3524191

[B58] Trimble W. S. Grinstein S. (2007). TB or not TB: calcium regulation in mycobacterial survival. Cell. 130, 12–14. 10.1016/j.cell.2007.06.039 17632049

[B59] Tufariello J. M. Chan J. Flynn J. L. (2003). Latent tuberculosis: mechanisms of host and bacillus that contribute to persistent infection. Lancet Infect. Dis. 3, 578–590. 10.1016/S1473-3099(03)00741-2 12954564

[B60] Tundup S. Pathak N. Ramanadham M. Mukhopadhyay S. Murthy K. J. Ehtesham N. Z.. (2008). The co-operonic PE25/PPE41 protein complex of *Mycobacterium tuberculosis* elicits increased humoral and cell mediated immune response. PLoS ONE. 3:e3586. 10.1371/journal.pone.0003586 18974870 PMC2570489

[B61] Vandal O. H. Nathan C. Ehrt S. (2009). Acid resistance in *Mycobacterium tuberculosis*. J. Bacteriol. 191, 4714–4721. 10.1128/JB.00305-09 19465648 PMC2715723

[B62] Vergne I. Chua J. Deretic V. (2003). Tuberculosis toxin blocking phagosome maturation inhibits a novel Ca2+/calmodulin-PI3K hVPS34 cascade. J. Exp. Med. 198, 653–659. 10.1084/jem.20030527 12925680 PMC2194170

[B63] Vergne I. Chua J. Singh S. B. Deretic V. (2004). Cell biology of *Mycobacterium tuberculosis* phagosome. Annu. Rev. Cell Dev. Biol. 20, 367–394. 10.1146/annurev.cellbio.20.010403.114015 15473845

[B64] WHO (2019). WHO Tuberculosis Report 2019. Geneva: World Health Organization.

[B65] Wickstrum J. R. Bokhari S. M. Fischer J. L. Pinson D. M. Yeh H. Horvat R. T. Parmely M. J. (2009). Francisella tularensis induces extensive caspase-3 activation and apoptotic cell death in the tissues of infected mice. Infect. Immun. 77, 4827–4836. 10.1128/IAI.00246-09 19703976 PMC2772556

[B66] Wijeratne S. S. K. Cuppett S. L. Schlegel V. (2005). Hydrogen peroxide induced oxidative stress damage and antioxidant enzyme response in caco-2 human colon cells. J. Agric. Food Chem. 53, 8768–8774. 10.1021/jf0512003 16248583

[B67] Yeruva V. C. Kulkarni A. Khandelwal R. Sharma Y. Raghunand T. R. (2016). The PE_PGRS proteins of *Mycobacterium tuberculosis* are Ca(2+) binding mediators of host-pathogen interaction. Biochemistry 55, 4675–4687. 10.1021/acs.biochem.6b00289 27483162

