# Supplementary figures and images for: PGRS Domain of Rv0297 of Mycobacterium tuberculosis Is Involved in Modulation of Macrophage Functions to Favor Bacterial Persistence
Source: Front Cell Infect Microbiol. 2020 Sep 11;10:451. doi: 10.3389/fcimb.2020.00451 (PMC7517703; doi:10.3389/fcimb.2020.00451)

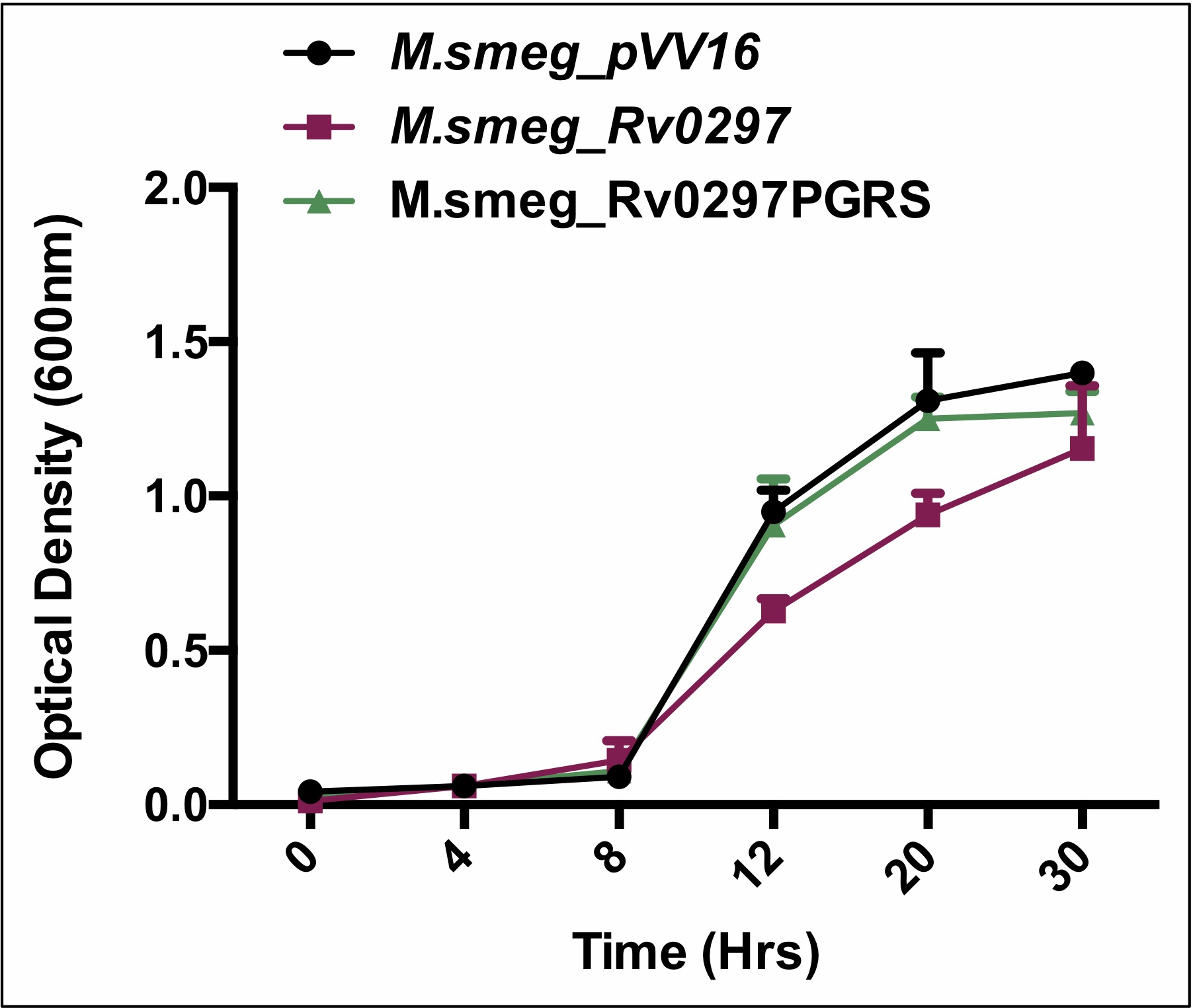

Supplement: Supplementary Figure 1 — Growth curve of recombinant M. smegmatis expressing full length Rv0297 or its PGRS domain. Growth curve analysis showed that expression of Rv0297 is not affceting the in vitro growth of recombinant bacterium. [file Image_1.JPEG]
